# Pax6 Directly Down-Regulates *Pcsk1n* Expression Thereby Regulating PC1/3 Dependent Proinsulin Processing

**DOI:** 10.1371/journal.pone.0046934

**Published:** 2012-10-09

**Authors:** Ting Liu, Yanxia Zhao, Na Tang, Ruopeng Feng, Xiaolong Yang, Nicole Lu, Jinhua Wen, Lingsong Li

**Affiliations:** 1 Peking University Stem Cell Research Center, and Department of Cell Biology, School of Basic Medical Sciences, Peking University Health Science Center, Beijing, China; 2 SARI Center for Stem Cell and Nanomedicine, Shanghai Advanced Research Institute, CAS, Shanghai, China; Joslin Diabetes Center, Harvard Medical School, United States of America

## Abstract

**Background:**

Heterozygous *paired box6* (*Pax6*) mutations lead to abnormal glucose metabolism in mice older than 6 months as well as in human beings. Our previous study found that *Pax6* deficiency caused down-expression of *prohormone convertase 1/3* (*Pcsk1*), resulting in defective proinsulin processing. As a protein cleaving enzyme, in addition to its expression, the activity of PC1/3 is closely related to its function. We therefore hypothesize that *Pax6* mutation alters the activity of PC1/3, which affects proinsulin processing.

**Methodology/Principal Findings:**

Using quantitative RT-PCR, western blot and enzyme assay, we found that PC1/3 C-terminal cleavage and its activity were compromised in *Pax6* R266Stop mutant mice, and the expression of *Pcsk1n*, a potent inhibitor of PC1/3, was elevated by *Pax6* deficiency in the mutant mice and MIN6 cells. We confirmed the effect of proSAAS, the protein encoded by *Pcsk1n*, on PC1/3 C-terminal cleavage and its activity by *Pcsk1n* RNAi in MIN6 cells. Furthermore, by luciferase-reporter analysis, chromatin immunoprecipitation, and electrophoretic mobility shift assay, we revealed that Pax6 bound to *Pcsk1n* promoter and directly down-regulated its expression. Finally, by co-transfecting *Pax6* siRNA with *Pcsk1n* siRNA, we showed that *Pax6* knock-down inhibited proinsulin processing and that this effect could be rescued by proSAAS down-regulation. These findings confirm that Pax6 regulates proinsulin processing partially through proSAAS-mediated PC1/3 processing and activity.

**Conclusions/Significance:**

Collectively, the above experiments demonstrate that Pax6 can directly down-regulate *Pcsk1n* expression, which negatively affects PC1/3 C-terminal cleavage and activity and subsequently participates in proinsulin processing. We identified *proSAAS* as a novel down-regulated target of Pax6 in the regulation of glucose metabolism. This study also provides a complete molecular mechanism for the *Pax6* deficiency-caused diabetes.

## Introduction

Paired box 6 (Pax6) is a transcription factor and a member of the paired-box gene family [1], [2]. It plays a critical role in regulating the development of the eyes, central nervous system and pancreas [3], [4], [5], [6]. The *Pax6*-null mouse exhibits defects in multiple organs and therefore cannot survive after birth [4], [7], [8]. In addition, mice with heterozygous *Pax6* mutations develop diabetes [6], [7], suggesting Pax6 also regulates glucose metabolism.

Using conditional gene-inactivation approach, people showed that Pax6 was actually not required for the specification, formation, or survival of pancreatic beta cells. Instead, it is essential for the late stage differentiation and maturation of pancreatic beta cells [9]. In the previous report, we found that Pax6 directly regulated the expression of prohormone convertase 1/3 (PC1/3) encoded by the gene *Pcsk1*, a serine protease essential for proinsulin processing. The down-regulation of *Pcsk1* led to defective proinsulin processing and abnormal glucose metabolism in *Pax6* mutant mice and in *PAX6*-deficient patients as well [10]. Mice with a disruption of *Pcsk1* exhibited growth retardation and several hormone precursors processing defects including proinsulin [11], [12]. This conclusion was further supported by a human pedigree study, which reported that the *PCSK1* mutation exhibited phenotypes including, but not limited to severe obesity, abnormal glucose homeostasis, elevated plasma proinsulin, low insulin level [13]. Human *PCSK1* deficiency also caused severe malabsorptive refractory neonatal diarrhea and shared most phenotypes with the first case including reactive hypoglycemia, and elevated circulating levels of certain prohormones [14].

ProSAAS, the protein encoded by *Pcsk1n*, is a potent inhibitor of PC1/3 [15], [16], [17]. ProSAAS is expressed by broad neuronal distribution in the central nervous system, while in the periphery it is an excellent marker of endocrine cells [18]. ProSAAS was found responsible for inhibition of PC1/3 activity and C-terminal cleavage [15], [16], [17], [19]. As reported, the activity of 66 kDa PC1/3 can be 40-folds higher than that of 87 kDa PC1/3, thus C-terminal cleavage was considered an important factor affecting the enzyme activity of PC1/3 [20]. Since there was an elevated fasting blood glucose level in adult *Pcsk1n* transgenic mice and impaired glucose tolerance in the *Cpe^fat/fat^* background transgenic mice [21], we sought to investigate whether the *Pcsk1n* expression is altered in the *Pax6* mutant mice, and if so, whether the alteration would affect PC1/3 C-terminal cleavage and activity, thereby contributing to the abnormal proinsulin processing and onset of diabetes in these *Pax6* mutant mice. In addition, to confirm the repressive function of Pax6 in regulation of gene expression as proposed by others [22], [23], [24], we also investigated whether Pax6 direct down-regulation of ProSAAS expression.

## Results

**Figure 1:**
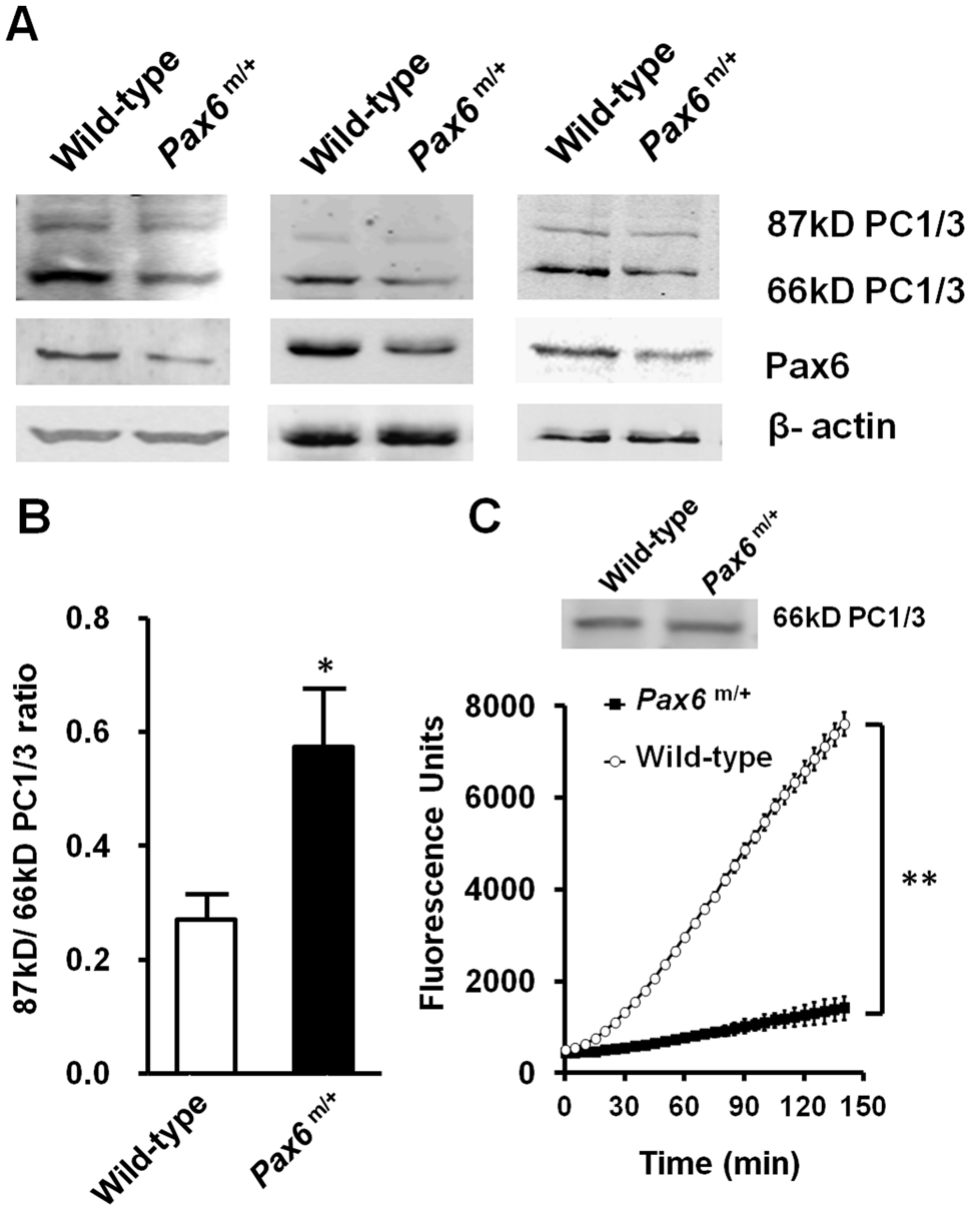
*Pax6* deficiency led to decrease of PC1/3 C-terminal cleavage and activity in mice. **A**, expression of 87 kDa and 66 kDa PC1/3 in isolated islets in three operations as measured by western blot, each blot show the islets protein from several mice in wild type (from left to right, *n* = 7, 3, 3) versus *Pax6* mutant mice (*n* = 9, 3, 3). 60 µg islets protein of wild-type and *Pax6* mutant mice was used. **B**, ratio of densitometry scanning of 87 kDa and 66 kDa PC1/3 bands is shown (n = 3). **C**, *Pax6* mutant mice (*Pax6^m/+^*) exhibited lowered activity of PC1/3. Islets protein of wild-type (*n* = 6) and *Pax6* mutant mice (*n* = 6) with equivalent PC1/3 normalized by western blot (the upper row) were used to determine PC1/3 activity in the presence of 400 µM fluorogenic substrate. Wild type: white bars, *Pax6^m/+^*: black bars. A representative experiment is shown; the experiment was repeated three times. Each value represents the mean ± SE. *p<0.05, **p<0.01.

We isolated pancreatic islets from either *Pax6* R266Stop mutants or normal sibling mice, and measured PC1/3 activity and its C-terminal processing. Data from 3 experiments of western blot analysis is shown in Fig. 1 A. The 87 kDa/66 kDa PC1/3 ratio was elevated in the islets of the *Pax6* mutant mice shown in Fig. 1 B. The results indicate that the mutant mice have defective PC1/3 processing in *Pax6* mutated islets. In addition to investigating PC1/3 C-terminal cleavage, we also investigated the activity of PC1/3 in the islets of the *Pax6* mutant mice. Considering the previously discovered fact that the expression of PC1/3 will be reduced in the islets of the mutant mice [10], the change of PC1/3 activity should be measured only when the amount of PC1/3 is equivalent. When the same amount of cleaved PC1/3 was used in enzyme activity analysis (Fig. 1C, upper row), the activity of PC1/3 was decreased in the mutant mice, as shown in Fig. 1 C. ProSAAS was reported to play roles in inhibiting PC1/3 activity and decreasing C-terminal cleavage of PC1/3 [19]. Therefore, our results indicate that proSAAS may be involved in the regulation of PC1/3 activity in *Pax6* mutant mice.

**Figure 2:**
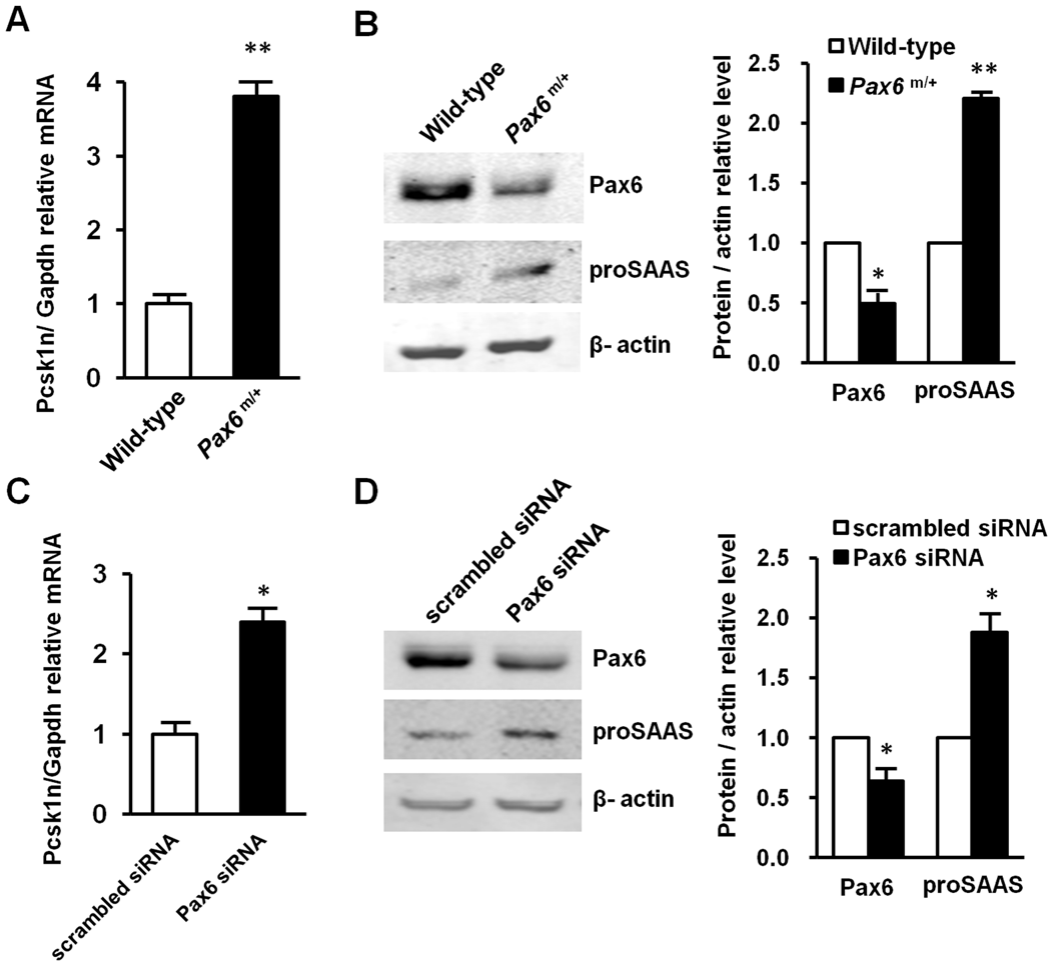
Decreased Pax6 led to increased *Pcsk1n* expression. **A**, **B** *Pax6* deficiency led to *Pcsk1n* up-regulation in the islets of *Pax6* R266Stop mutant mice. *Pcsk1n* expression was detected by qRT-PCR (**A**) (Wild type: white bars, *Pax6^m/+^*: black bars; n = 3) or by western blotting analysis (**B**). *Pcsk1n* expression level in islets isolated from *Pax6* mutant mice at age of 6 months (*n* = 9) were significantly higher than those in wild-type mice (*n* = 7). Protein quantification ratio of densitometry scanning of *Pax6* ^m/+/^Wild-type was shown right. **C**, **D** Pax6 knock-down elevated *Pcsk1n* expression in MIN6 cells. *Pcsk1n* expression was detected in MIN6 cells with *Pax6* knock-down by qRT-PCR (**C**) (n = 3) or by western blotting analysis (**D**) (n = 3). *Pcsk1n* expression levels in MIN6 cells transfected with *Pax6* siRNA (black bars) or scrambled siRNA (white bars) were identified. Quantification ratio of densitometry scanning of protein transfected withPax6 siRNA/scrambled siRNA was shown right. A representative experiment is shown; the experiment was repeated three times. Each value represents the mean ± SE. *p<0.05, **p<0.01.

To investigate whether the expression of *Pcsk1n* was affected in the pancreatic islets of the *Pax6* mutant mice, we measured *Pcsk1n* expression by qRT-PCR and western blot. Indeed, *Pcsk1n* was significantly elevated in the pancreatic islets of *Pax6* mutant mice as measured by qRT-PCR (Fig. 2A) and western blot (Fig. 2B) in comparison with the results of the pancreatic islets of normal mice, while Pax6 was decreased. Furthermore, both *Pcsk1n* mRNA (Fig. 2C) and proSAAS proteins (Fig. 2D) were significantly elevated upon the decrease in Pax6-knocked down in MIN6 cells. The graphs in Fig. 2B and 2D were quantification for scanning western blot bands of Pax6 and proSAAS relative to β-actin, respectively. These observations showed that decreased Pax6 led to an increase of proSAAS production in the pancreatic islets of *Pax6* mutant mice and in MIN6 cells, which indicates Pax6 down-regulates *Pcsk1n* expression in islets.

**Figure 3:**
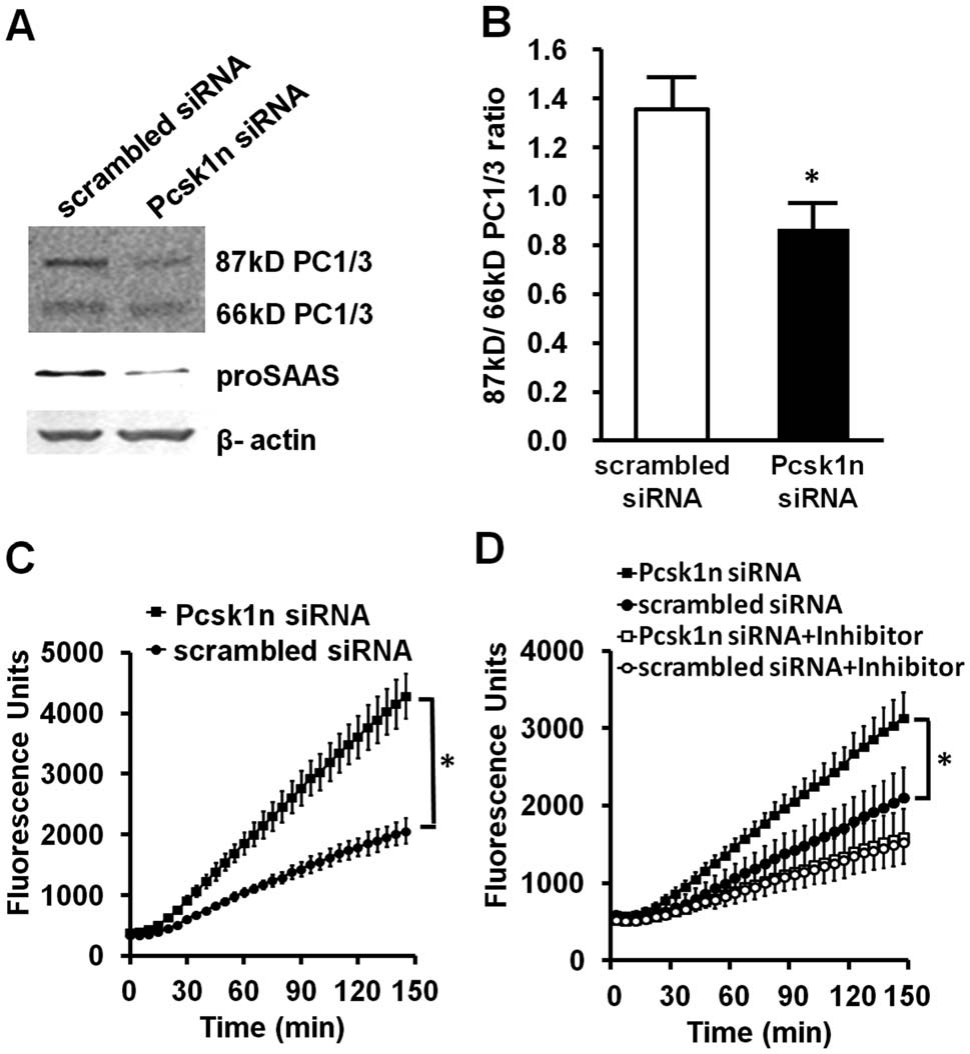
Down-regulation of *Pcsk1n* advanced C-terminal cleavage and activity of PC1/3 in MIN6 cells. **A**, MIN6 cells were transfected with *Pcsk1n* siRNA for 48 h, and protein was collected to analyze the PC1/3 C-terminal cleavage with western blots. **B**, ratio of densitometry scanning of 87 kDa and 66 kDa PC1/3 bands was analyzed using MIN6 cells transfected with *Pcsk1n* siRNA (black bar) or scrambled siRNA (white bar) (n = 3). **C**, enzyme activity was analyzed using MIN6 cells transfected with *Pcsk1n* siRNA (black circles) or scrambled siRNA (white circles) with equivalent protein (n = 3). **D**, the specificity of enzyme activity measured was tested by PC1/3 specific inhibitor Ac-LLRVKR-NH_2_ using MIN6 cells. Black squares, *Pcsk1n* siRNA; Black circles, scrambled siRNA; White squares, *Pcsk1n* siRNA + inhibitor; White circles, scrambled siRNA + inhibitor. A representative experiment is shown; the experiment was repeated three times. Each value represents the mean ± SE. *p<0.05.

To investigate the possibility that the decrease of proSAAS promotes PC1/3 processing and activity, we transfected *Pcsk1n* siRNA in MIN6 cells. Results showed that the 87 kDa/66 kDa PC1/3 ratio had decreased (Fig. 3 A and B), contrary to its increase in *Pax6* mutant mice. In Fig. 3A & 3B the knock-down of *Pcsk1n* was shown to affect the cleavage of PC1/3 pro-form. Fig. 3C shows that Pcsk1n not only affects the cleavage of PC1/3 pro-form but also the activity of PC1/3. When the final amount of 66 kD PC1/3 was the same, PC1/3 activity was increased in *Pcsk1n* knock-down cells. This observation indicates that proSAAS actually inhibits the activity of PC1/3 by affecting its cleavage as well as its enzyme activity. Because the substrate used in the enzyme assay can be hydrolyzed not only by PC1/3 but also by PC2 and furin, it is necessary to investigate the specificity of the enzyme assay. The PC1/3 specific inhibitor Ac-LLRVKR-NH_2_, a peptide derived from proSAAS, was added to the enzyme assay reaction system according to Apletalina et.al and Cameron et.al [25], [26]. As shown in Fig. 3D, Ac-LLRVKR-NH2 dramatically blocked the enzyme activity to a lower level with Pcsk1n and scrambled siRNA. This is because Ac-LLRVKR-NH_2_ almost blocked all the PC1/3 activity, but with scrambled siRNA, some PC1/3 activity remained. The blocked enzyme activity shows that PC1/3 plays a major role in the enzyme activity assay system. This observation indicates that reduced enzyme activity in the islets of *Pax6* mutants is mainly due to the deficiency of PC1/3 activity and the elevation of its inhibitor proSAAS. We also observe diminished PC1/3 expression level after *Pcsk1n* knock-down, which is consistent with reports that proSAAS co-expression increases synthesis and secretion of PC1/3 in CHO cells [19]. However, whether or not Pax6 affected PC1/3 C-terminal cleavage and its activity through regulating the *Pcsk1n* expression level remains to be revealed.

**Figure 4:**
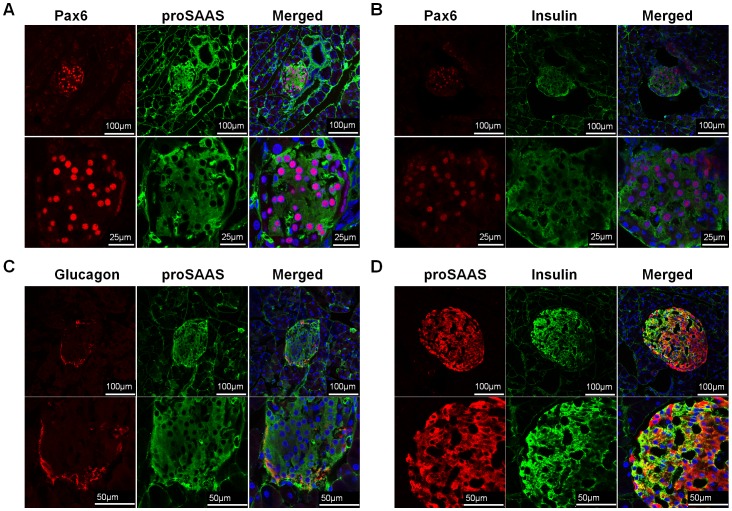
Pax6 and proSAAS were co-localized in β cell of pancreatic islets. Immunofluorescent staining for Pax6 and proSAAS (**A**), Pax6 and insulin (**B**), Glucagon and proSAAS (**C**), and proSAAS and insulin (**D**) in the pancreas of mice. Blue, DAPI.

**Figure 5:**
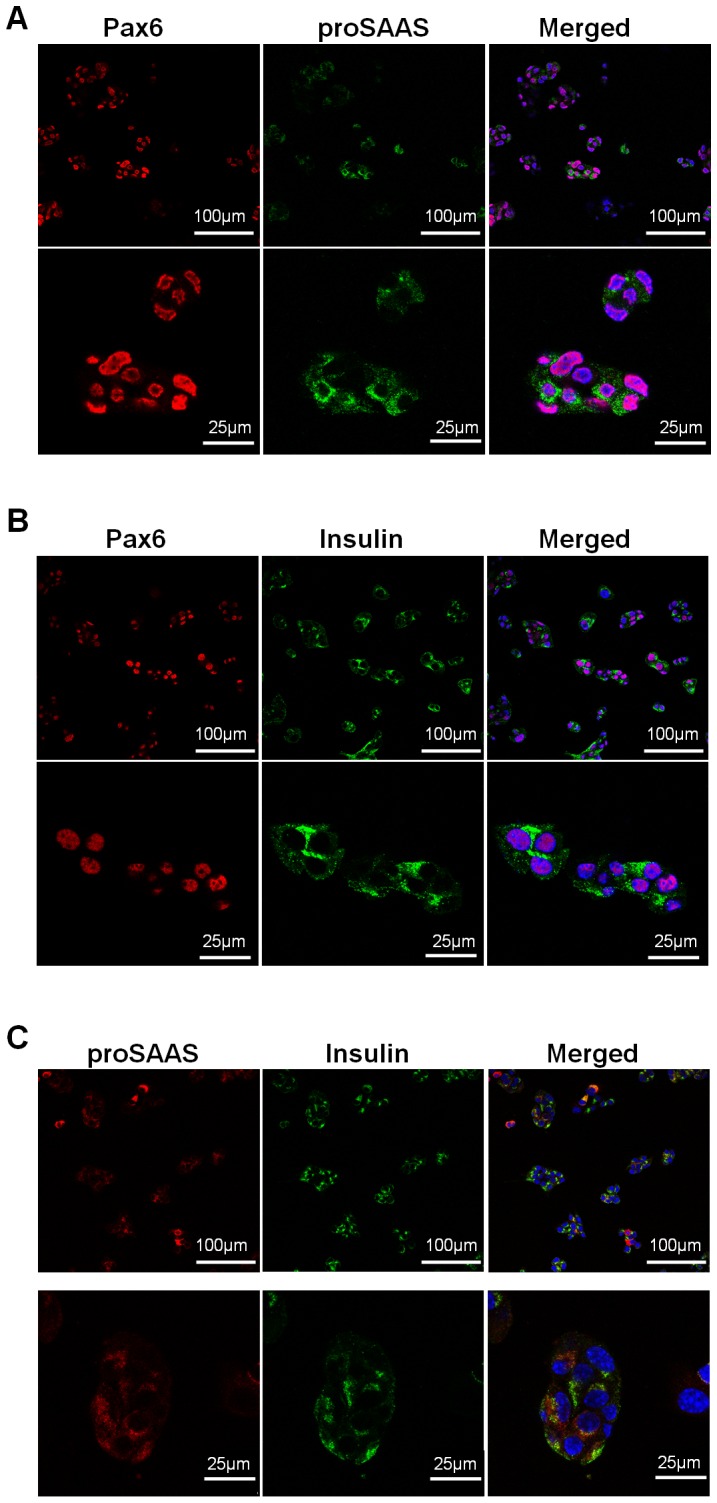
Pax6 and proSAAS were expressed in MIN6 cells. Immunofluorescence staining for Pax6 and proSAAS (**A**), Pax6 and insulin (**B**) and proSAAS and insulin (**C**) in MIN6 cells. Blue, DAPI.

To answer this question, we first analyzed the distribution of proSAAS and Pax6 in the pancreatic islets as well as in MIN6 cells by immunofluorescence staining. The results indicated that not only Pax6 co-localized with proSAAS (Fig. 4A & 5A), but Pax6 also co-localized with insulin in pancreatic islets and MIN6 cells (Fig 4 B & 5B). Furthermore, we investigated the co-localization of proSAAS and endocrine hormones, such as insulin and glucagon. ProSAAS was expressed in β-cells (Fig 4 D, 5C), though some α-cells was also stained with proSAAS (Fig 4 C). We noticed that in β-cells the signal of insulin was lower as the signal of proSAAS was higher and vice versa (Fig 4 D). Similar results were observed in MIN6 cells (Fig. 5C). This observation showed that the expression of proSAAS might get involved in down-regulating insulin processing.

**Figure 6:**
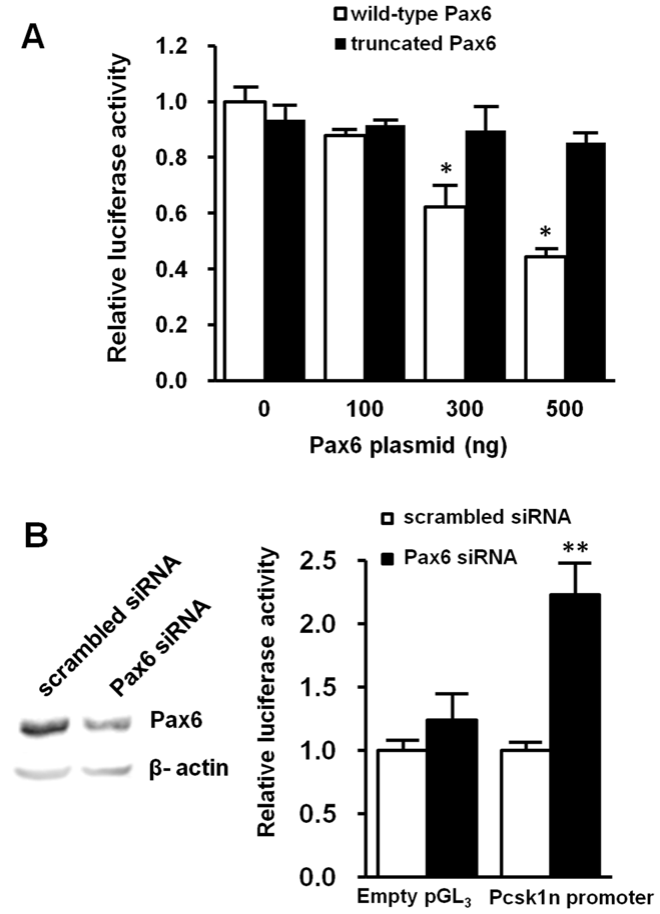
Pax6 directly down-regulated *Pcsk1n* expression. **A**, Wild-type Pax6 (white bars), but not the truncated Pax6 (black bars), down-regulated *Pcsk1n* promoter activity as analyzed by using a luciferase report system in HEK293 cells (n = 3). **B**, in MIN6 cells transfected with Pax6 siRNA (black bars) but not scrambled siRNA (white bars) led to an increase in luciferase activity of the −1999Luc promoter construct (indicated as *Pcsk1n* promoter) (n = 3). Results of empty pGL_3_ vectors are showed as a negative control. Decreased Pax6 production in cells after transfecting Pax6 siRNA was confirmed by western blotting analysis (upper panel). Each value represents the mean ± SE. *p<0.05, **p<0.01.

**Figure 7:**
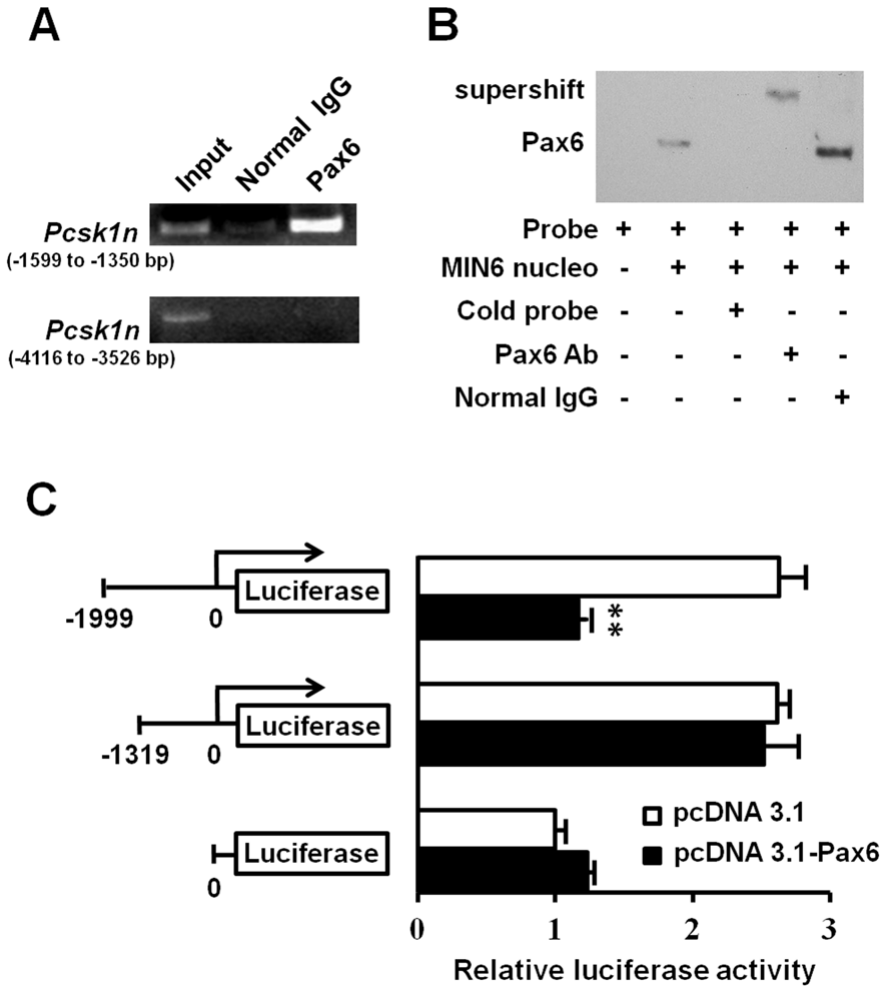
Pax6 bound to the promoter region of *Pcsk1n* and down regulated its expression. **A**, Pax6 was recruited on the *Pcsk1n* promoter. Soluble chromatin from MIN6 cells was immunoprecipitated with Pax6 antibody or a rabbit normal IgG. The final DNA extractions were amplified by PCR using primers that cover the upstream control region (lower panel) or the proximal promoter region of the *Pcsk1n* gene (upper panel). **B**, analyses of the binding site of Pax6 to the *Pcsk1n* promoter by EMSA. EMSA was performed with nuclear extracts of MIN6 cells. Specific competitor (cold probe, lane 3) was used as a control. For supershift, antibody to Pax6 (lane 4) or rabbit normal IgG (lane 5) was incubated with nuclear extracts before added to the reaction. Arrows indicate the shifted or supershifted band of protein-DNA complexes. **C**, Pax6 down-regulated the activity of full-length promoter of *Pcsk1n*, and had no effect on 5′-truncated *Pcsk1n* promoter or empty pGl_3_ luciferase activities in HEK293 cells (n = 3). White bars, pcDNA3.1(-) empty plasmid control; black bars, *Pax6* expressive plasmid. Each value represents the mean ± SE **p<0.01.

To test the hypothesis that Pax6 regulates *Pcsk1n* expression in islets, we first performed a luciferase reporter assay using *Pcsk1n* promoter activity reporter system. Wild-type Pax6 but not the R266Stop mutation moiety led to a dose-dependent decrease of *Pcsk1n* promoter activity in HEK293 cells (Fig. 6A); knock-down of *Pax6* led to elevation of *Pcsk1n* promoter activity in MIN6 cells (Fig. 6 B).To identify the Pax6 binding site in the *Pcsk1n* promoter, we investigated a 2000 bp up-stream sequence of *Pcsk1n* promoter from the transcription start site, as pGl_3_-full-length promoter, based on Genomatix MatInspector database [27]. The bioinformatics analysis results suggested a putative binding site: sequences from −1433 to −1415 bp containing a Pax6 core binding sequence *TTCATGCTTG* [28] from the transcriptional start site. Based on the ChIP analyses, Pax6 was able to bind to the *Pcsk1n* promoter within (−1599 to −1350 bp) region (Fig. 7 A, upper panel). This binding was specific since the other regions including the sequence of −4116 to −3526 bp did not show Pax6 binding (Fig. 7 A, lower panel).

To further certify the binding, a long oligonucleotide containing the candidate binding site of the sequence from −1433 to −1415 bp was used as a labeled probe in an EMSA. When the labeled oligonucleotide representing the promoter sequence from −1441 to −1408 bp was incubated with nuclear extract prepared from MIN6 cells, a protein-probe complex was detected. When 100-fold molar excess of competitive unlabeled DNA was added, the formation of protein-probe complex failed (Fig. 7B). Moreover, the addition of Pax6-specific antibody, but not the normal IgG control, caused a supershift band (Fig. 7B). These findings indicate the Pax6 binding site is located at 33 base pairs within the region of −1441 to −1408 bp from the transcriptional start site of the *Pcsk1n* promoter. This is consistent with the bioinformatics analysis.

To functionally confirm this Pax6 binding site for regulation of *Pcsk1n* expression, we performed truncated luciferase reporter analysis for *Pcsk1n* promoter activity. *Pcsk1n* transcriptional activity was found significantly decreased when Pax6 was over-expressed using pGl_3_-full-length promoter system. On the other hand, Pax6 no longer affected the *Pcsk1n* promoter activity when the region of −999 to −1319 bp was deleted (Fig. 7C).

**Figure 8:**
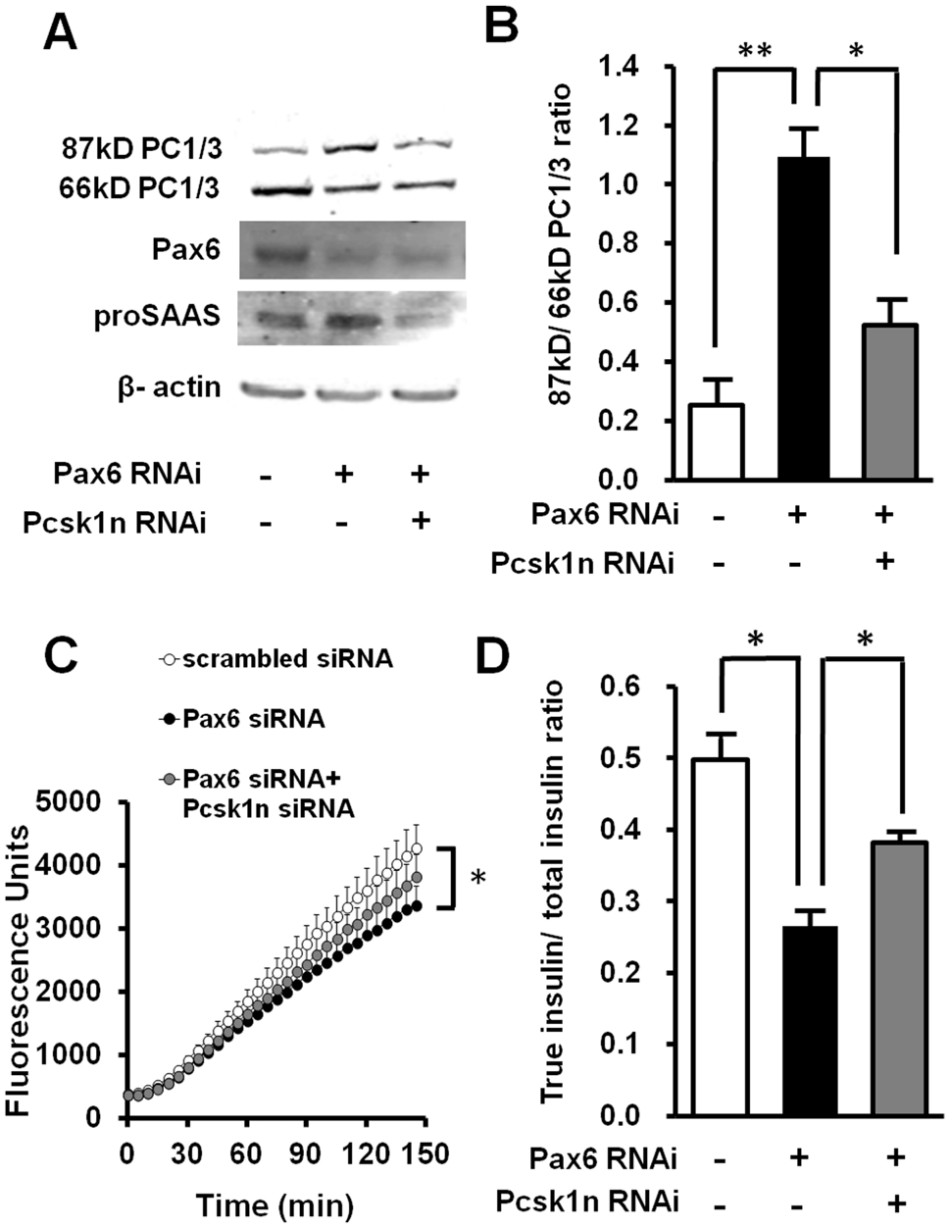
Down-regulationof *Pcsk1n* rescued the effect of Pax6 knock-down on PC1/3 and proinsulin processing. **A**, MIN6 cells were transfected with *Pax6*, *Pax6* and *Pcsk1n* or scrambled siRNA in triplicate, and 48 hours later protein was analyzed by western blot. **B**, ratio of densitometry scanning of 87 kDa and 66 kDa PC1/3 bands is shown (n = 3). **C**, enzyme activity of PC1/3 was examined in *Pax6* RNAi or *Pax6* and *Pcsk1n* double RNAi treated MIN6 cells with equivalent protein. Scrambled siRNA was used as negative control (n = 3). Black circles, values for transfected with Pax6 siRNA; Grey circles, values for transfected with both *Pax6* and *Pcsk1n* siRNA; White circles, scrambled siRNA. **D**, the true insulin and total insulin concentrations in conditional medium of MIN6 cells were measured by ELISA. The cells were transfected with scrambled siRNA, Pax6 siRNA or *Pax6* and *Pcsk1n* siRNA, and aliquots of conditional medium were applied to ELISA Kits for total insulin or true insulin detection (n = 3). True insulin, the mature form of insulin, which was derived from proinsulin cleaved by PC1/3 and PC2; total insulin, the sum of true insulin and proinsulin. White bars, value for transfected with scrambled siRNA; Black bars, value for transfected with Pax6 siRNA; Grey bars, value for transfected with both of *Pax6* and *Pcsk1n* siRNA. A representative experiment is shown; the experiment was repeated three times. Each value represents the mean ± SE. *p<0.05, **p<0.01.

Knock-down of *Pax6* led to up-regulation of proSAAS (Fig. 2, Fig. 8A). Knock-down of *Pcsk1n* elevated PC1/3 C-terminal cleavage and activity (Fig. 3, Fig 8 A, B and C). This is consistent with the fact that PC1/3 C-terminal cleavage and activity in *Pax6* mutant pancreatic islets is decreased (Fig. 1). In addition to *Pax6* RNAi, *Pcsk1n* and *Pax6* double knock-down could decrease the proSAAS production (Fig. 8A), and partially rescue the decreased PC1/3 C-terminal cleavage (Fig. 8A and B) and activity (Fig. 8C) induced by *Pax6* knock-down alone. Since PC1/3 C-terminal cleavage and activity is closely related to proinsulin processing, as indicated by true insulin/total insulin ratio, we further investigated the concentration of true insulin and total insulin in conditional medium of MIN6 cells by ELISA. The double knock down of *Pax6* and *Pcsk1n* partially recovered the true insulin/total insulin ratio, compared with that of cells transfected with just *Pax6* siRNA (Fig. 8D).

## Discussion

Using *Pax6* heterozygous R266Stop mutant mice as an animal model, we showed in previous study that Pax6 regulated glucose metabolism via up-regulation of PC1/3, an essential enzyme for proinsulin processing. In this follow-up study, we revealed that C-terminal cleavage and activity of PC1/3 were also compromised in pancreatic islets of *Pax6* mutant mice. On the other hand proSAAS, a protein responsible for inhibition of PC1/3 C-terminal cleavage [19] and activity [15], [16], [17], [19], was significantly increased in *Pax6* mutant mice. We further proved that Pax6 bound directly to the *Pcsk1n* promoter and down-regulated *Pcsk1n* expression. Jointly, the above results reveal a more complete mechanism for *Pax6* deficiency related diabetes. Specifically, Pax6 affects proinsulin processing and glucose metabolism through up-regulation of PC1/3 and down-regulation of proSAAS. This study also identifies proSAAS as a mediator between Pax6 and PC1/3 in pancreatic islets.

It has been reported that over-expression of proSAAS reduced the rate of processing of the endogenous prohormone proopiomelanocortin at a PC1/3-specific site [16]. Our study proves that proSAAS also regulates proinsulin processing via inhibiting C-terminal cleavage and activity of PC1/3 in pancreatic cells for the first time. It has been reported that *Pcsk1n* transgenic mice exhibited elevated fasting serum glucose [21], which supports the finding that proSAAS functions as a proinsulin processing related molecule in blood glucose regulation. The study of fetal mice with a *Pcsk1n* mutation suggests that proSAAS-derived peptides could inhibit PC1/3 in an embryonic brain, but the PC1/3 activity was not affected by the absence of *Pcsk1n* in adult mice [29]. This finding in neuropeptide processing is not consistent with our results in islets. Our study focuses on proinsulin processing in islets of *Pax6* mutant mice, in which the expression of *Pcsk1n* is elevated. Differing from the *Pcsk1n* mutant mice reported by Morgan et al., our finding is more consistent with the impaired fasting glucose of *Pcsk1n* transgenic mice [21]. In support of our findings, Fortenberryet.al has also found that full-length proSAAS inhibits PC1/3 activity in CHO/PC1/3 cells [19]. It seems that in the central nerve system, proSAAS functions as neuropeptides regulating body weight and other behaviors, but in the endocrine system, proSAAS regulates PC1/3 maturation and activity, though its regulation seems to be modest. Relatively little research has focused on proSAAS’s endocrinal functions, and the reason for its distinguished roles remains to be revealed.

Our previous study has reported that the proinsulin/insulin ratios in *Pax6* mutant mice older than 6 months were significantly higher than those in wild-type controls. These mice exhibit impaired glucose tolerance. In this study, the *Pax6* mutant mice at the age of 4 months showed elevated *Pcsk1n* expression. The aging related regulation suggests an effect of the accumulation of heterozygous mutation in mice.

In α cells of pancreatic islets Pax6 up-regulates PC2 and its inhibitory chaperon 7B2 [30] and in β cells we find Pax6 up-regulates PC1/3 but down-regulates *Pcsk1n*. *Pcsk1n* derived peptides show structural and functional homology with 7B2 [15], [19], and this difference in regulation by Pax6 may correspond with different functions of proSAAS and 7B2. Although both proSAAS and 7B2 potently inhibit PCs, their intracellular interactions with PCs differ significantly. The N-terminal domain of proSAAS has no effect on stabilization of PC1/3 activity, different from the effect of the N-terminal domain of 7B2 on PC2 [19].

While most research reveals an up-regulation effect of Pax6, we identify a novel down-regulated target of Pax6, proSAAS, which participates in the regulation of proinsulin processing and glucose metabolism. There have been a few down-regulated targets of Pax6 identified. VEGFA, a growth factor and MMP2, a cellular matrix protein, involved in tumor formation and metastasis are found repressed by Pax6 [22], [23]. Olig2, a transcription factor critical for glial cell fate determination during forebrain development, is also directly repressed by Pax6 [24].Our finding of the down regulation of *Pcsk1n* by Pax6 provides a potential target for improving proinsulin processing and attenuating *Pax6* deficiency-caused abnormal glucose metabolism.

The role of Pax6 in proinsulin processing has been constantly followed by other researchers. For instance, Gosmainet.al has found that Pax6 represents a key component of the transcriptional network in α cells [31] and β cells [32], regulating a series of molecules including PC1/3. This result supports our findings above. In summary, our study reveals a novel regulating pathway of Pax6-proSAAS-PC1/3 activity in proinsulin processing as part of glucose metabolism regulation.

## Materials and Methods

### Cell Lines, Constructions and Transfection

The HEK293 cell line was obtained from American Type Culture Collection (ATCC, Manassas, VA, USA). The MIN6 cell line was a generous gift from Dr. Guang Ning (Department of Endocrinology and Metabolism, Ruijin Hospital, Shanghai Jiao Tong University School of Medicine, Shanghai Clinical Center for Endocrine and Metabolism Diseases, Endocrine and Metabolic E-Institutes of Shanghai Universities and Key Laboratory for Endocrinology and Metabolism of Chinese Health Ministry, Shanghai, People’s Republic of China). The MIN6 cells derived from pancreatic beta-cell tumors [33] were maintained in DMEM/High Glucose with 15% (vol./vol.) Fetal bovine serum (FBS) and 50 µmol/l β-mercaptoethanol. The HEK293 cells were maintained in DMEM/High Glucose with 10% (vol./vol.) FBS. All cell culture media was obtained from Gibco (Gaithersburg, MD, USA), FBS from Hyclone (Thermo Fisher Scientific, Waltham, MA, USA) and β-mercaptoethanol from Gibco (Gaithersburg, MD, USA). The Scrambled siRNA(Cat. No. AM4611 or AM4613), siRNAs for *Pax6* (siRNA ID: s71268 or s71269) and siRNAs for *Pcsk1n* (siRNA ID: s203223 or s78146) were from Ambion (Austin, TX, USA), and all RNAi results were confirmed with different siRNA IDs. Cells were transiently transfected with plasmids using lipofectamine 2000 (Invitrogen, Carlsbad, CA, USA) or siRNA using lipofectamine RNAiMAX (Invitrogen, Carlsbad, CA, USA) following the manufacturer’s protocols.

## Animals

Animals using were as previously described (11). Briefly, a stop-condon mutation at 266 aa of Pax6 was led by injecting the chemical mutagen N-ethyl-N-nitrosourea. After breeding with normal mice, the mutation was transmitted to newborns. Based on the small-eye phenotype, mice with heterozygous *Pax6* gene mutation can be selected. Mice were maintained in a pathogen-free facility under a 12 h light–dark cycle and were given free access to food and water. This study was carried out in strict accordance with the recommendations in the Guide for the Care and Use of Laboratory Animals of the National Institutes of Health [34]. The protocol was approved by the Peking University animal ethics committee, and all efforts were made to minimize suffering. All data relevant not specifically mentioned were collected after 6 months of birth.

### Isolation and Purification of Mouse Pancreatic Islets

Mouse pancreatic islets were isolated and purified following Hori’s method [35]. Briefly, pancreases were digested with type V collagenase (Sigma-Aldrich, St Louis, MO, USA), and centrifuged (2,700 g) in PBS. Then individual islets were handpicked under a dissecting microscope, without bringing attached acinar, vascular or ductal tissues.

### Immunofluorescence

Frozen sections of pancreas or MIN6 cells fixed in 4% paraformaldehyde were immunostained with either normal rabbit and mouse IgG, or rabbit antibody to Pax6 (MBL, Naka-ku Nagoya, Japan), rabbit antibody to proSAAS (Abcam, Cambridge, UK), mouse monoclonal antibody to proSAAS (Abnova, Taipei, Taiwan), rabbit antibody to glucagon (Zhongshanjinqiao, Beijing, China), mouse monoclonal antibody to insulin (Zhongshanjinqiao, Beijing, China) overnight at room temperature, and then with mounting medium (DAPI; Vector Laboratories, Burlingame, CA, USA).

### Quantitative RT-PCR

RNA of pancreatic islets or cells was extracted with RNeasy Micro Kit (Qiagen, Valencia, CA, USA) and reversed to cDNA. The primers used in quantitative RT-PCR (qRT-PCR) were, for *Pax6*: forward primer 5′-ACCCGGCAGAAGATCGTAG-3′, reverse primer 5′-TTTGCATCTGCATGGGTCT-3′; for *Pcsk1n*: forward primer 5′- GTGGACCCTGAGCTGCTG-3′, reverse primer 5′- AAATCCTGGTCCACAGATCG-3′; for Gapdh as a control: forward primer 5′-CGTGCCGCCTGGAGAAACCTG-3′, reverse primer 5′-AGAGTGGGAGTTGCTGTTGAAGTCG-3′.

### Western Blot

The protein of pancreatic islets or cells was separated by 10% (wt/vol.) SDS-PAGE and probed with antibodies including rabbit PAX6-specific antibody (Abcam, Cambridge, UK), rabbit proSAAS-specific antibody (Sigma-Aldrich, St Louis, MO, USA), rabbit PC1/3-specific antibody (Abcam, Cambridge, UK), and mouse β-actin-specific monoclonal antibody (Sigma-Aldrich, St Louis, MO, USA).

### Enzyme Assays

Enzyme assays were conducted using 400 µmol/l Pyr-Glu-Arg-Thr-Lys-Arg-methylcoumarin amide (Peptide Institute, Osaka, Japan) as a substrate in an assay previously described [36]. Protein of islets or MIN6 cells were subjected with substrate in 100 mmol/l sodium acetate, pH 5.0, containing 5 mmol/l CaCl_2_, 0.1% Triton X-100, and a protease inhibitor mixture composed of 1 µM *trans*-epoxysuccinic acid, 1 µmol/l pepstatin, 280 µmol/l TPCK, and 140 µmol/l TLCK. All assays were performed at 37°C in a 96-well plate fluorometer with an excitation wavelength of 380 nm and an emission wavelength of 460 nm. The total volume was 50 µl. When used, 120 nM of PC1/3 specific inhibitor Ac-LLRVKR-NH_2_ (synthesized by Bioss. Inc, Beijing, China) was pre-incubated with protein at room temperature for 30 min prior to the addition of the substrate.The activity was measured duplicate or triplicate every 5 min for a period of 2.5 h, and the rates of hydrolysis over this time were calculated; the first 10 min were excluded from the rate calculation. Enzyme activity is given in fluorescence units per minute (FU/min) where one FU corresponds to 8.6 pmol of methylcoumarin amide.

### Luciferase Reporter Assay

Mouse *Pcsk1n* promoter (−1999 bp to 0 bp) and serial deletions were cloned from mouse genomic DNA and inserted into pGL_3_-promoter luciferase reporter vectors. The primers were listed as follows: forward primer for −1999Luc (−1999 bp to 0 bp) 5′-CGAGCTCAATCCTCTGGGTTTGGTGCTGA-3′, for -1319Luc (-1319 bp to 0 bp) 5′-CGAGCTCACCTTAAACTTATGACCCTCCT-3′, reverse primer for both 5′-TCCCCCGGGCCCCCTCCCCGCGTCCCCGTCC-3′. Pax6 was cloned and inserted into pcDNA3.1 (−) vectors. HEK293 or MIN6 cells were cultivated in 24 well plates for 24 hours and transfected with mouse *Pcsk1n* promoters or pGL_3_-promoter vector along with pcDNA3.1 (−) vector, Pax6 cDNA or truncated Pax6 cDNA (expressing the moiety as in the *Pax6* R266Stop mutant) at several dosages. Cells were collected 48 h after transfection and assayed in triplicate with the Dual-Luciferase Reporter Assay System (Promega, Madison, WI, USA).

### Chromatin Immunoprecipitation Assays

Chromatin immunoprecipitation (ChIP) assays were performed in MIN6 cells. Briefly, the chemical-linked complexes of genomic DNA and Pax6 protein were immunoprecipitated with antibodies specific to Pax6 (Chemicon, Rosemont, Illinois, USA). The DNA was amplified with LA taq (TAKARA, Shiga, Japan) after ChIP using primers of *Pcsk1n* promoter −1599∼−1350 bp (forward primer 5′-TGTGCCATCTCTCCAGCCCAGC-3′, reverse primer 5′-ATCTAGCAGCTAGATAATGTCT-3′), and −4116∼−3526 bp (forward primer 5′-TAGACCAGGATGGCCTCAAACTAAAA-3′, reverse primer 5′-AGCCCTAAACCCTTACTTCTGACCT-3′.

### Electrophoretic Mobility Shift Assay

Electrophoretic Mobility Shift Assay (EMSA) was performed with LightShift Chemiluminescent EMSA Kit (Pierce, Rockford, USA) following the manufacturer’s protocols. Briefly, 5 micrograms of nuclear extract from MIN6 cells were added in a reaction mixture. The oligonucleotides used were: 5′-TTTCTTGGTGAACTCCAAGCATGAACGTGCTCTA-3′. Probes were synthesized with biotin labeled at the 5′ terminal and then annealed. For supershift experiments, nuclear extracts were preincubated with antibody for 10 min on ice before the addition of biotin-labeled DNA probe and subsequently incubated at room temperature for 10 min. Competition was carried out with a 100-fold excess of the unlabeled probe. All reaction mixtures were loaded onto a 6% nondenaturing polyacrylamide gel, and after electrophoresis, the gel was exposed to X-ray film for 1 min.

### ELISA

MIN6 cells at required time point were washed twice with PBS and once with DMEM-High, and incubated with 1 mL DMEM-High per well (6 well plate) for 4 h. The conditioned medium was centrifuged and appropriately diluted, and 10 µl aliquots were subjected for ELISA. True insulin and total insulin were measured with an ELISA kit from Shibayagi Company (Ishihara Shibukawa-shi, Gumma, Japan) according to the manufacturer’s instructions. True insulin is the mature form of insulin that was cleaved by PC1/3 and PC2, and total insulin is the sum of true insulin and proinsulin. Thus the ratio of true insulin/total insulin indicates proinsulin processing. Since the cross-reactivity of true insulin and proinsulin is less than 5% according to the manufacturer’s description, the calculated ratios of true insulin over total insulin in mice are likely to be not precise, but are acceptable for the present purposes of comparison.

### Statistical Analysis

Statistical analysis was performed using an independent samples t test. Differences were considered statistically significant at *p*<0.05.
